# Predictors of 30-day readmission among those treated with alcohol withdrawal in acute hospitals in England

**DOI:** 10.1093/alcalc/agaf022

**Published:** 2025-05-09

**Authors:** Thomas Phillips, Rachel Coleman, Simon Coulton

**Affiliations:** Centre for Addiction and Mental Health Research (CAMHR), University of Hull, East Yorkshire, Cottingham Road, Hull HU6 7RX, United Kingdom; Alcohol Care Team, Department of Gastroenterology, Hull Royal Infirmary, Hull University Teaching Hospitals NHS Trust, East Yorkshire, Anlaby Road, Hull HU3 2JZ, United Kingdom; Centre for Addiction and Mental Health Research (CAMHR), University of Hull, East Yorkshire, Cottingham Road, Hull HU6 7RX, United Kingdom; Centre for Addiction and Mental Health Research (CAMHR), University of Hull, East Yorkshire, Cottingham Road, Hull HU6 7RX, United Kingdom; Centre for Health Services Research, University of Kent, Kent, Canterbury CT2 7NF, United Kingdom

**Keywords:** alcohol dependence, alcohol withdrawal, acute hospitals, readmission

## Abstract

**Aims:**

To examine predictors of 30-day readmissions to acute hospitals in England for patients treated for alcohol withdrawal (AW).

**Methods:**

Retrospective cross-sectional analysis of routine hospital administrative data (i.e. Hospital Episode Statistics—Admitted Patient Care records) for adults admitted to non-specialist hospitals in England 2017–18.

**Results:**

AW admissions were associated with digestive, circulatory, respiratory, and endocrine disorders and were of short duration (median 3 days). Of the 19 588 completed AW admissions examined in 2017–18, 3957 (20.2%) resulted in readmission within 30 days. The strongest predictors of 30-day readmission were being no fixed abode (Adjusted Odds Ratio (AOR) 1.81, 95%CI 1.44–2.26), prior discharge against medical advice (AOR 1.57, 95%CI 1.40–1.77), and greater Charlson comorbidity index total score (AOR 1.02, 95%CI 1.02–1.03).

**Discussion:**

AW 30-day admissions are common and associated to complex case presentations that require high levels of community support on discharge. Hospital-based alcohol teams should prioritize strategies, which maximize medically managed AW, effective transitions to specialist community care including outreach teams and strong collaborations with physical and mental health outpatient services. Together with specialist initiatives within community mental health teams, assertive outreach, and homeless services 30-day readmissions may be minimized.

## Introduction

It is currently estimated that ~600 000 adults in England experience alcohol dependence (Pryce et al. 2017), with only 13% of the in-need population accessing specialist alcohol services per annum the increasing rise in alcohol-related hospital admissions has been associated to limited access to specialist inpatient treatment (Phillips et al. 2021). Over the last decade, alcohol admissions to non-specialist hospitals in England have continued to rise (PHE 2023), current estimates suggest that 1 in 10 adults admitted are experiencing alcohol dependence (Roberts et al. 2019).

Internationally, the clinical and financial impacts of alcohol disorders on non-specialist settings have led to the development of hospital-based alcohol services to improve the identification, treatment and through care of patients with the aim of enhancing outcomes and reducing the demand on hospital services (NHS England 2019, Medicare Learning Network 2024). Those admitted with alcohol withdrawal (AW) (ICD-10 code F10.3) often experience suboptimal care within non-specialist services. AW is triggered by the sudden cessation or reduction of alcohol use in those with physical dependence, and if not adequately treated may progress to severe complications (i.e. seizures or delirium tremens) or even death. Abstinence from alcohol is the predominant treatment goal following the management of AW requiring the support of specialist treatment interventions and a network of support. Previous studies have identified those admitted to non-specialist services with AW commonly have a *cluster of clinical complexity* (Phillips et al. 2019, Blackwood et al. 2021, Roberts et al. 2021), receive negative treatment experiences (Simon et al. 2020), and a lack of follow-up care (Neighbors et al. 2005). These factors appear to contribute to a lack of engagement in community interventions following discharge and subsequent repeated hospital admissions; however, few studies in the UK have specifically examined the direct relationship between AW and readmission rates in non-specialist hospitals. No similar study has been previously conducted using a national representative dataset in England. Understanding the characteristics and predictors of 30-day readmission following an AW admission is essential to ensure hospital-based alcohol care teams develop appropriate clinical strategies that target those most likely to be readmitted. This study therefore aims to explore the characteristics and predictors of AW readmissions using Hospital Episode Statistics—Admitted Patient Care (HES-APC) records, which are routinely collected by the NHS in England.

## Materials and methods

We conducted a cross-sectional retrospective analysis using 2017–18 HES-APC records for England, which provides a national representative sample of clinical activity in non-specialist hospitals. Ethical approval for the study was obtained from the Faculty of Health Sciences, University of Hull (Ref: FHS180). An extract including all adult (18 years or more) admissions from 1 April 2017 to 31 March 2018 was purchased from the data custodians NHS Digital for the purpose of the study (DARS-NIC-226185-B6C2J) and included over 9.3 million Finished Consultant Episodes (FCEs) experienced by 5.3 million adults. These data included demographic information, activity data, and diagnostic codes using ICD-10 (WHO 1992). A FCE is the period admitted under the care of a single consultant. Each hospital stay contains one or more FCE to create a hospital spell of care (i.e. admission). A unique HES-ID allows the number of admissions to be assigned to an individual, calculation of the length of stay (LOS) for each admission and where appropriate the time in days between admissions. Each FCE provides up to 20 ICD-10 diagnostic codes, which were searched using the *regexm* command in STATA 15SE (Stata Corp.) to identify cases admitted including AW, additional ICD-10 diagnoses to calculate the Charlson comorbidity index (CCI) score (NHS Digital 2019, Charlson et al. 2022).

### Selection of cases

A conservative approach was adopted where cases were selected based on the presence of the ICD-10 F10.3 code as a primary or secondary diagnosis in any FCE of an admission in the first eleven months of the year. A 30-day readmission (i.e. ≤30 days) for any reason was calculated for an individual to identify whether a hospital admission resulted in a subsequent readmission ≤30 days from date of discharge. We excluded from analysis admissions related to a patient who died in hospital, or where the consultant episode remained open after the 1 March 2018.

### Characteristics and predictors of AW readmission

Demographic variables including age, sex, and ethnicity were considered for inclusion. Whilst age and sex were fully coded, ethnicity codes were poorly completed. Missing at random analysis was conducted on the completeness of ethnicity codes, which identified that these cases were missing not at random (*P* < .001). Multiple imputation was subsequently attempted to reduce the number of missing cases in the model and compared with removing ethnicity from the model altogether. We determined that there was little difference in imputing ethnicity data and removing it when considering predictors of readmission. Therefore, the results of this paper focus on the model with ethnicity data removed.

Indices of multiple deprivation (IMD) scores were available in the dataset; however, this was not included in the model as those who were coded as having no fixed abode (NFA) did not have an IMD value recorded due to a lack of a postcode. NFA was therefore used as a dichotomous variable to indicate deprivation in the population and within the model.

Other variables in the dataset considered for inclusion in the model were emergency versus planned admission (including planned, booked, or elective), day of admission, and discharge and LOS. LOS was considered as a continuous variable calculated from admission and discharge dates with those admitted for <24 hours coded as LOS 1 day. LOS of 11 days or more was recoded as 11+ to avoid the influence of additional days unrelated to AW management as guidelines suggest initial medical treatment for AW rarely exceeds 10 days (NICE 2010).

### Statistical analyses

Analysis was conducted for all AW admissions in the dataset, with comparisons between those where a readmission in 30 days occurred, and those where there was no subsequent readmission in 30 days.

Once the dataset had been prepared, and cases selected, associations between characteristics were examined to determine the relationship between factors and those readmitted within 30 days, and those which were not. This exploration incorporated demographic characteristics (age, sex, ethnicity, living circumstances), features of admissions (admission method, day of the week of admission), comorbidity (number of ICD-10 diagnoses on admission, ICD-10 diagnosis codes by chapter and CCI score), LOS (total days admitted), and features of discharge (day of the week of discharge, discharge against medical advice [DAMA]).

Two-way tables and Chi-squared tests were used to examine differences between groups for dichotomous variables, with non-parametric tests employed to examine differences between means for continuous variables. Where necessary categorical variables were transformed into dichotomous variables to allow for exploration of differences and associations using Chi-squared tests. Where the association between the variable and readmission in 30 days had a statistical significance level of *P* = ≤.15, the variable was included in the logistic regression model to explore predictors of readmission.

Logistic regression was used to generate crude odds ratios for individual variables, and adjusted odds ratios within the logistic regression model. The HES data extract included AW admissions from 195 hospital providers. We controlled for hospital provider by incorporating the provider variable into our regression model, to reduce the potential impact of medical coding bias from each provider.

The *vif* command in STATA 15 SE was used to explore multi-collinearity within the model, where a value of 1 indicates no correlation between a given variable and any other variables in the model, a value of 1–5 indicates a moderate correlation, and a value >5 indicates a potentially severe correlation.

## Results

The HES-APC dataset contained 21 493 individuals who had a combined total of 27 444 AW admissions within 2017–18. Once those who had died or were still open <30 days at year end were removed, there were 19 588 completed AW admissions in year of which 3957 (20.2%) of completed AW admissions resulted in readmission within 30 days. The mean age was significantly higher in the readmitted group and those in the readmitted group were more likely to be male and living NFA (Table 1). Almost all methods of admission were categorized as emergency admissions with very few non-emergency admissions (i.e. planned, booked, or elective) identified as involving a diagnosis of AW. No significant association between admission method or day of the week admitted were identified between groups.

**Table 1:** Study sample characteristics and variables associated with readmission in 30 days.

		Readmitted within 30 days	Not readmitted within 30 days	*P*-value
Number (% of sample)		3957 (20.2)	15 631 (79.8)	
Age				
Mean age in years		50.1	49.7	.035^a^
(95% CI)		(49.7–5.5)	(49.5–49.9)	
Sex				
Male (% col)		2940 (74.3)	11 300 (72.3)	.012^a^
Ethnicity (Caucasian vs. non-Caucasian)				
Caucasian (% col)		3451 (93.3)	13 298 (92.5)	.125^b^
No fixed abode (NFA) (NFA vs. not NFA)				
Living NFA (% col)		139 (3.5)	311 (2.0)	<.001^c^
Admission method (emergency vs. not emergency)				
Emergency (% col)		3851 (97.3)	15 209 (97.3)	.913
Weekend vs. weekday admission				
Weekend (% col)		976 (24.7)	3856 (24.7)	.996
Charlson comorbidity index (CCI)				
Mean CCI total score		4.7	3.7	<.001^c^
(95%CI)		(4.5–4.9)	(3.6–3.8)	
CCI category				
CCI score = 0 (*n*, % col)		2142 (54.1)	9498 (60.8)	
CCI score = 1–5 (*n*, % col)		693 (17.5)	2612 (16.7)	
CCI score ≥ 5 (*n*, % col)		1122 (28.4)	3521 (22.5)	
Length of stay				
Mean number of days		4.6	4.5	.113^b^
(95%CI)		(4.5–4.7)	(4.4–4.6)	
Median number of days		3	3	.734
(Inter-quartile range)		(1–8)	(1–7)	
Day of discharge (% col)				
Day 1		1080 (27.3)	4565 (29.2)	
Day 2		526 (13.3)	1890 (12.1)	
Day 3		382 (9.7)	1413 (9.0)	
Day 4		276 (7.0)	1235 (7.9)	
Day 5		257 (6.5)	1131 (7.2)	
Day 6		252 (6.4)	940 (6.0)	
Day 7		191 (4.8)	794 (5.1)	
Day 8		152 (3.8)	504 (3.2)	
Day 9		83 (2.1)	355 (2.3)	
Day 10		88 (2.2)	351 (2.2)	
Day 11+		670 (16.9)	2453 (15.7)	
Weekend vs. weekday discharge				
Weekend (% col)		651 (16.5)	2539 (16.2)	.751
Discharge against medical advice (yes vs. no)				
Yes (% col)		548 (13.9)	1485 (9.5)	<.001^c^

^a^Statistically significant at .05 level.

^b^Statistically significant at .15 level.

^c^Statistically significant at .01 level.

Greater clinical comorbidity measured by mean CCI total score was significantly associated with 30-day readmission, with 28.4% those in the 30-day readmitted group scoring CCI > 5 compared to 22.5% in those not readmitted. The distribution of clinical diagnosis for each admission group for all 22 ICD-10 chapter headings is provided in Supplementary Table S1.

No statistical association was found between LOS and 30-day readmission, with 58% of all AW admissions being completed in <5 days and with the overall mean LOS (4.52 days; 95%CI 4.47–4.57) and median LOS (3 days; IQR 1–7) suggesting a suboptimal treatment period for the completion of AW across those admitted.

Whilst the day of discharge was not associated with readmissions within a shorter period time, DAMA was significantly associated with readmission in 30 days, with 13.91% of those in the readmitted group having discharged against medical advice, compared to 9.46% of those not readmitted within 30 days.

### Predictors of readmission in 30 days

The strongest predictors of 30-day readmission of those previously admitted with AW were being NFA (AOR 1.81, 95%CI 1.44–2.26), DAMA (AOR 1.57, 95%CI 1.40–1.77), and greater CCI total score (AOR 1.02, 95%CI 1.02–1.03) (Table 2). The multiple-imputation model which synthesized imputed data for ethnicity additionally identified being male was also predictive of 30-day readmission among those previously admitted with AW (Supplementary Table S2).

**Table 2:** Adjusted^a^ odds ratios for 30-day readmission for patients admitted to hospitals in England with alcohol withdrawal during 2017–18.

Variable	Adjusted odds ratios (95%CI)	*P*-value
No fixed abode	1.81 (1.44–2.26)	<.001^b^
Discharge against medical advice	1.57 (1.40–1.77)	<.001^b^
Sex—Male	1.08 (0.97–1.20)	.151
CCI total score	1.02 (1.02–1.03)	<.001^b^
Length of stay	1.00 (0.99–1.02)	.532
Age	1.00 (1.00–1.01)	.675

^a^Adjusted for hospital provider within the regression model.

^b^Statistically significant at .01 level.

The *vif* command in STATA 15 SE was used to explore any further multi-collinearity in the model. Variance inflation factor values ranged from 1.04 to 1.36, therefore indicating an acceptable level of correlation between each variable and other variables in the final regression model.

## Discussion

To our knowledge, this is the first study to examine 30-day readmission rates among adults experiencing AW during non-specialist hospital admissions in England using nationally representative routine data. Our results identify 20% of those identified as experiencing AW are readmitted within 30 days of discharge from hospital, with the three main predictors of readmission being individuals of NFA, self-discharge, or DAMA from hospital and those experiencing greater clinical comorbidity as measured by CCI scores. Previous analysis of hospital admissions in England from 2004 to 2010 reports that the readmission rate for the wider hospital population (experiencing all diagnoses) is 7% (Blunt et al. 2014).

The largest previous study examining the impact of AW readmissions within non-specialist hospitals was conducted in the USA and identified similar outcomes. Yedlapati and Stewart (2018) conducted a cross-sectional retrospective evaluation of AW readmission rates and predictors of 30-day AW readmissions using a 2013 national representative sample in acute care and identified *n* = 393 118 AW discharges. The authors identified a 30-day readmission rate of 19.7% (95%CI 19.0–20.4) and found DAMA and comorbid psychotic disorder as the strongest predictors of 30-day readmissions. Similar findings were identified in a study examining 30-day readmissions using the 2014 US Readmissions Database for all alcohol-related disorders (ARDs), which identified a readmission rate of 18.9% with those with ARD and comorbid conditions at greater risk of early readmission (Wani et al. 2019).

Our analysis identified the strongest predictor of 30-day readmission to be NFA which is a key determinant of health linked to increasing healthcare use due to complex and untreated clinical comorbidities including mental health, substance use disorders, and excessive alcohol use (Fountain et al. 2003). Arguably, admissions of those with NFA are potentially preventable if housing issues are addressed; however, individuals legitimately admitted due to clinical concerns involving AW are likely to lack the support required to establish abstinence on discharge. Given the short length of admission (mean of 4.6 days) appropriate accommodation is unlikely to be secured placing the individual at a high risk of returning to dependent drinking that exacerbates preexisting conditions resulting in early readmission.

DAMA has previously been linked to increased mortality (Southern et al. 2012) and higher 30-day readmission rates than planned discharges (Spooner et al. 2020). Our previous analysis (Coleman et al. 2023) examined DAMA against those experiencing planned discharges for those treated for AW and identified emergency admissions, shorter LOS (2.7 vs. 4.7 days, *P* < .001) and weekend discharges as characteristics significantly associated with DAMA.

All individuals experienced a complex cluster of clinical conditions with an average of eight diagnosed conditions, with digestive, circulatory, respiratory, and endocrine disorders among the most recorded comorbid conditions. Whilst we have observed greater comorbidity is predictive of 30-day readmission, we are unable to ascertain if these readmissions are preventable if effective alcohol treatment is provided on discharge or anticipated due the unpredictable nature and severity of these comorbid conditions. Previous analysis of routine hospital data in England (Blunt et al. 2014), which examined all 30-day readmissions, estimated that only 30% are potentially preventable as the majority experienced legitimate deteriorating medical presentations and were identified as unavoidable or ‘anticipated’ 30-day readmissions.

Although policy intentions focus on the need to reduce alcohol-related admissions as a measure of effectiveness of services, it remains to be established to what extent AW readmissions can be preventable due to the high levels of comorbidity experienced by those admitted. Our analysis points toward the care of AW and transition initial recovery from alcohol dependence is likely to be suboptimal given the median LOS of 3 days means that many individuals may experience discharge at the peak of AW with limited time to facilitate meaningful transitions to specialist community services, outpatient services, or housing where needed.

### Strengths and limitations

The use of routine administrative data provides a comprehensive national understanding as to how AW readmissions impact non-specialist hospitals, the scale of which is not possible to obtain from prospective observational studies. Additionally, the use of routine administrative data allows for the inclusion of cases who are often excluded from surveys due to poor health or inability to provide informed consent. The methods employed by medical coders, who analyse patient’s records, to endorse specific discharge clinical diagnoses means the attribution of AW is highly reliable as only emergent clinical sequalae of AW will be recorded by clinicians.

The findings from this study should be interpreted considering the following limitations. Routine administrative data are used for commissioning purposes in England and Wales, and therefore not collected in accordance with research principles, although the quality of these datasets has improved over time (Aylin et al. 2007). Phillips et al. (2019) identified those with any alcohol diagnosis as accounting for 9.2% of hospital admissions using previous HES-APC data, whereas Roberts et al. (2019) systematic review and meta-analysis identified a prevalence of 19.76% for harmful drinking and 10.25% for alcohol dependence in admitted adults in England, indicating that HES data provide a conservative estimate of need when compared to routine alcohol screening. Whilst HES data may under report alcohol-specific admissions, AW presentations are more likely than other forms of alcohol-related presentations to be classified correctly as the coding is based on observable clinical symptoms of withdrawal. The introduction of the NHS long-term plan has resulted in greater investment in hospital-based alcohol care teams since 2019 (NHS England 2019) with the aim of introducing hospital-wide routine alcohol screening. It is therefore possible that the introduction of routine and systematic screening of AUD and AW will increase the detection of cases and influence the recorded rate of those readmitted 30 days; however, it is unclear whether the assessments of hospital-based alcohol services will increase accuracy and frequency of coding within the HES-APC dataset. Missing data related to ethnicity are a concern as this inhibits our understanding of health inequalities and health outcomes for different ethnic groups. The work addressing consistency of ethnicity data recorded in health-related administrative datasets including HES-APC being undertaken by the Office of National Statistics (ONS 2024) should increase our understanding of alcohol interventions across all groups.

### Implications for future research

Future research should build on the findings of this study to reduce 30-day readmission rates in patients experiencing AW. Community-based (e.g. general practice, district nursing) datasets could be linked to HES data to explore patient journeys and variables preceding or associated with 30-day readmission to strengthen the model developed here. Introduction of universal routine screening in acute hospitals will identify higher numbers of individuals at risk of AW which may indicate an initial increase in cost to the NHS; however, prospective studies should be designed to assess this and explore overall reduction in alcohol-related admissions, DAMA, and 30-day readmissions. Observational and prospective research should consider quality of treatment including level of prescribing of medication to treat AW symptoms. Qualitative work should be undertaken to explore staff and patient perspectives and data on reasons for DAMA and 30-day readmissions to acute hospitals.

## Conclusion

In conclusion, this analysis identifies implications for the alcohol treatment system in England, which during the same financial year identified 4720 individuals admitted to specialist alcohol inpatient care for AW (NDTMS 2025). Greater treatment capacity and access to specialist inpatient care and collaborative relationships between non-specialist and specialist provision is required to address the impact of emergency admissions requiring treatment for AW and subsequent readmission to hospital within 30 days. Hospital-based alcohol liaison teams should aim to maximize the effectiveness of medically managed AW, consider active collaboration with specialist community service prior to discharge, but importantly support those subspecialist services such as community diabetic services, outpatient cardiology, and liver services and strong collaborations with community mental health teams, assertive outreach, and homeless services are essential to tailor care.

## Supplementary Material

Supplementary_Table_agaf022

Supplementary_Table_2_26-04-25_agaf022

## Data Availability

The data underlying this analysis was supplied by NHS Digital (now NHS England) using a data agreement which prevents sharing of the raw data. This data is however available via the Data Access Request Service (DARS) application at https://digital.nhs.uk/services/data-access-request-service-dars
