# Supplementary material for: Predictors of 30-day readmission among those treated with alcohol withdrawal in acute hospitals in England
Source: Alcohol Alcohol. 2025 May 9;60(3):agaf022. doi: 10.1093/alcalc/agaf022 (PMC12063994; doi:10.1093/alcalc/agaf022)
Supplement: Supplementary_Table_agaf022 [file supplementary_table_agaf022.docx]

Supplementary Table 1. ICD10 diagnoses associated with readmission in 30 days for patients admitted with AW in hospitals in England in 2017/2018

|  |  | **Readmitted within 30 Days** | **Not Readmitted within 30 Days** | **P-value** |
| --- | --- | --- | --- | --- |
| **Number (% of sample)** |  | 3,957 (20.2) | 15,631 (79.8) |  |
| **ICD-10 Diagnoses on Admission** | | | | |
| Mean Number of Diagnoses |  | 8.2 | 7.6 | ** <0.001 |
| (95% CI) |  | (8.1-8.3) | (7.6-7.7) |  |
| **ICD10 Chapter 1. Infectious & Parasitic Diseases** | | | | |
| Yes (% col) |  | 482 (12.2) | 1,548 (9.9) | ** <0.001 |
| **ICD10 Chapter 2. Neoplasms** | | | | |
| Yes (% col) |  | 124 (3.1) | 375 (2.4) | ** 0.009 |
| **ICD10 Chapter 3. Diseases of the blood** | | | | |
| Yes (% col) |  | 380 (9.6) | 1,430 (9.15) | 0.378 |
| **ICD10 Chapter 4. Endocrine, nutritional and metabolic diseases** | | | | |
| Yes (% col) |  | 1,143 (28.9) | 4,674 (29.9) | 0.211 |
| **ICD10 Chapter 5. Mental and behavioural disorders (exc. Alcohol)** | | | | |
| Yes (% col) |  | 25 (0.6) | 66 (0.4) | 0.083 |
| **ICD10 Chapter 6. Diseases of the nervous system** | | | | |
| Yes (% col) |  | 666 (16.8) | 2,306 (14.8) | ** 0.001 |
| **ICD10 Chapter 7. Diseases of the eye and adnexa** | | | | |
| Yes (% col) |  | 102 (2.6) | 424 (2.7) | 0.639 |
| **ICD10 Chapter 8. Diseases of the ear** | | | | |
| Yes (% col) |  | 37 (0.9) | 144 (0.9) | 0.935 |
| **ICD10 Chapter 9. Diseases of the circulatory system** | | | | |
| Yes (% col) |  | 1,383 (35.0) | 4,876 (31.2) | ** <0.001 |
| **ICD10 Chapter 10. Diseases of the respiratory system** | | | | |
| Yes (% col) |  | 1,107 (28.0) | 3,860 (24.7) | ** <0.001 |
| **ICD10 Chapter 11. Diseases of the digestive system** | | | | |
| Yes (% col) |  | 1,889 (47.7) | 6,595 (42.2) | ** <0.001 |
| **ICD10 Chapter 12. Diseases of the skin and subcutaneous tissue** | | | | |
| Yes (% col) |  | 331 (8.4) | 1,037 (6.6) | ** <0.001 |
| **ICD10 Chapter 13. Diseases of the musculoskeletal system and connective tissue** | | | | |
| Yes (% col) |  | 595 (15.0) | 2,074 (13.3) | ** 0.004 |
| **ICD10 Chapter 14. Diseases of the genitourinary system** | | | | |
| Yes (% col) |  | 503 (12.7) | 1,855 (11.9) | 0.145 |
| **ICD10 Chapter 15. Pregnancy, childbirth and the puerperium Indexed** | | | | |
| Yes (% col) |  | <5 cases | <10 cases | 0.735 |
| **ICD10 Chapter 17. Congenital malformations, deformations and chromosomal abnormalities** | | | | |
| Yes (% col) |  | 17 (0.4) | 67 (0.4) | 0.993 |
| **ICD10 Chapter 18. Symptoms, signs and abnormal clinical and lab findings not elsewhere classified** | | | | |
| Yes (% col) |  | 2,030 (51.3) | 7,727 (49.4) | * 0.036 |
| **ICD10 Chapter 19. Injury, poisoning and certain other consequences of external causes** | | | | |
| Yes (% col) |  | 863 (21.8) | 3,145 (20.1) | * 0.019 |
| **ICD10 Chapter 20. External causes of morbidity and mortality** | | | | |
| Yes (% col) |  | 400 (10.1) | 1,371 (8.8) | ** 0.009 |
| **ICD10 Chapter 21. Factors influencing health status and contact with health services** | | | | |
| Yes (% col) |  | 2,255 (57.0) | 8,086 (51.7) | ** <0.001 |
| **ICD10 Chapter 22. Codes for special purposes** | | | | |
| Yes (% col) |  | 26 (0.7) | 92 (0.6) | 0.619 |

**statistically significant at 0.01 level

Supplementary Table 2. Adjusted^†^ odds ratios for 30-day readmission for patients admitted to hospitals in England with alcohol withdrawal during 2017/18 – using multiple-imputation estimates for ethnicity

| **Variable** | **Adjusted Odds Ratios**  **(95% CI)** | **P-value** |
| --- | --- | --- |
|  |  |  |
| No Fixed Abode (NFA) | 1.81 (1.47-2.22) | **<0.001 |
| Discharge against medical advice (DAMA) | 1.57 (1.41-1.75) | **<0.001 |
| Ethnicity - Caucasian | 1.12 (0.96-1.30) | 0.156 |
| Sex – Male | 1.08 (1.00-1.17) | *0.049 |
| CCI Total Score | 1.02 (1.02-1.03) | **<0.001 |
| Length of Stay | 1.00 (0.99-1.01) | 0.465 |
| Age | 1.00 (1.00-1.00) | 0.731 |

^†^ Adjusted for hospital provider within the regression model

**statistically significant at 0.01 level
