# Supplementary material for: Predictors of 30-day readmission among those treated with alcohol withdrawal in acute hospitals in England
Source: Alcohol Alcohol. 2025 May 9;60(3):agaf022. doi: 10.1093/alcalc/agaf022 (PMC12063994; doi:10.1093/alcalc/agaf022)
Supplement: Supplementary_Table_2_26-04-25_agaf022 [file supplementary_table_2_26-04-25_agaf022.docx]

Supplementary Table 2. Adjusted^†^ odds ratios for 30-day readmission for patients admitted to hospitals in England with alcohol withdrawal during 2017/18 – using multiple-imputation estimates for ethnicity

| **Variable** | **Adjusted Odds Ratios**  **(95% CI)** | **P-value** |
| --- | --- | --- |
|  |  |  |
| No Fixed Abode (NFA) | 1.81 (1.47-2.22) | **<0.001 |
| Discharge against medical advice (DAMA) | 1.57 (1.41-1.75) | **<0.001 |
| Ethnicity - Caucasian | 1.12 (0.96-1.30) | 0.156 |
| Sex – Male | 1.08 (1.00-1.17) | *0.049 |
| CCI Total Score | 1.02 (1.02-1.03) | **<0.001 |
| Length of Stay | 1.00 (0.99-1.01) | 0.465 |
| Age | 1.00 (1.00-1.00) | 0.731 |

^†^ Adjusted for hospital provider within the regression model

**statistically significant at 0.01 level
